# Unsuppressible Repetition Suppression and exemplar-specific Expectation Suppression in the Fusiform Face Area

**DOI:** 10.1038/s41598-017-00243-3

**Published:** 2017-03-13

**Authors:** Auréliane Pajani, Sid Kouider, Paul Roux, Vincent de Gardelle

**Affiliations:** 1 Brain and Consciousness group (ENS, EHESS, CNRS), Département d’Études Cognitives, École Normale Supérieure - PSL Research University, 75005 Paris, France; 2 Service Universitaire de Psychiatrie d’adultes, Centre Hospitalier de Versailles, 78157 Le Chesnay, France; 3 Laboratoire HandiRESP, EA4047, UFR des sciences de la santé Simone Veil, Université Versailles Saint-Quentin-en-Yvelines, 78180 Montigny-le-Bretonneux, France; 4 Fondation Fondamental, Créteil, France; 5 Centre d’Economie de la Sorbonne, CNRS & Université Paris 1, 75013 Paris, France; 6 Paris School of Economics, 75014 Paris, France

## Abstract

Recent work casts Repetition Suppression (RS), i.e. the reduced neural response to repeated stimuli, as the consequence of reduced surprise for repeated inputs. This research, along with other studies documenting Expectation Suppression, i.e. reduced responses to expected stimuli, emphasizes the role of expectations and predictive codes in perception. Here, we use fMRI to further characterize the nature of predictive signals in the human brain. Prior to scanning, participants were implicitly exposed to associations within face pairs. Critically, we found that this resulted in exemplar-specific Expectation Suppression in the fusiform face-sensitive area (FFA): individual faces that could be predicted from the associations elicited reduced FFA responses, as compared to unpredictable faces. Thus, predictive signals in the FFA are specific to face exemplars, and not only generic to the category of face stimuli. In addition, we show that under such circumstances, the occurrence of surprising repetitions did not trigger enhanced brain responses, as had been recently hypothesized, but still suppressed responses, suggesting that repetition suppression might be partly ‘unsuppressible’. Repetition effects cannot be fully modulated by expectations, which supports the recent view that expectation and repetition effects rest on partially independent mechanisms. Altogether, our study sheds light on the nature of expectation signals along the perceptual system.

## Introduction

The reduced neural response to repeated stimuli, called Repetition Suppression (RS), has been robustly demonstrated in a wide range of experimental setups, from single-cell recordings in primates^1, 2^ to functional Magnetic Resonance Imaging (fMRI) in humans^3, 4^.

RS was initially considered as resulting from low-level and local adaptation mechanisms by which the response of a neuronal population would be reduced when a stimulus appears for the second time. This reduction could be obtained in several ways^5^. In brief, the fatigue model entails that all neurons that initially respond to a stimulus show a reduction in their response to the repetition of this stimulus, proportionally to their initial response, possibly through synaptic depression. In the facilitation model, by contrast, repeated inputs are processed faster and result in shorter latencies or shorter durations of neural firing, possibly through synaptic potentiation. In yet another model, the sharpening model, repeated inputs would be represented more sparsely, with stronger suppression in the neurons that code features irrelevant to stimulus recognition, possibly through inhibition from lateral connections within neuron populations.

However, recent years have seen the development of an alternative view linking RS to top-down expectations, within a predictive coding scheme. According to this scheme, the brain constantly predicts forthcoming inputs, and computes the discrepancy between actual and predicted sensory states, i.e. surprise, to update its predictions^6, 7^. Consistently with predictive coding, a growing body of evidence demonstrates that brain responses to expected inputs are reduced as compared to brain responses to unexpected inputs, a phenomenon termed “Expectation Suppression”^8–11^. This entails that if repeated inputs are less surprising than non-repeated ones, the surprise signal that they trigger in the corresponding sensory regions would be reduced through predictive mechanisms^12, 13^, thus resulting in RS. In line with this view, a number of recent brain imaging studies have shown that RS is modulated by repetition probability, with greater RS when repetitions are more likely^14–19^, although the conditions of occurrence of this modulation is still a matter of investigation^20–23^. Interestingly, the predictive coding framework might encompass the local adaptation accounts^24^, if one assumes that local adaptation is a specific implementation of one particular prediction: the prediction that stimuli would repeat in our environment. This prediction would result in suppressed responses to repeated inputs through predictive mechanisms.

Several questions arise from this perspective. How deeply is this general prediction about repetitions entrenched in the cortex? Can it be reversed? Could face-sensitive cortices form expectations about non-repeating face stimuli? The present study aims at addressing these questions.

It has been recently proposed that, in a context where repetitions are more surprising than non-repetitions (also called alternations), they should lead to enhanced brain responses instead of suppression^25^. Yet, previous studies testing repetitions that occurred with low probabilities (P = 0.25) did not find repetition enhancement, but found reduced RS^14–19^. A possible explanation is that in these studies, the use of novel, trial-unique faces during alternation trials might have created a situation in which repetitions were always easier to predict, even when they were less likely than alternations. Indeed, when presented with the first face of a trial, even if participants knew that an alternation was more likely than a repetition of this face, they could not predict which particular second face would occur in the case of an alternation. The current face would thus be, in the absence of a better prediction, the best guess for what would be presented next.

In the present study, we evaluated RS in a context in which repetitions were unlikely and where alternations were predictable, in order to investigate the direction of the repetition effects in this context (suppression vs. enhancement). In our main experimental condition (‘Prediction Blocks’), participants (N = 28) were exposed to a sequence of faces, using 3 pairs of associated faces, such that each face in a pair predicted the subsequent occurrence of its counterpart. Specifically, when a face appeared (Face1), it was very likely to be followed by its paired associate (Face2alt, p = 0.75), and much less likely to be followed by its own repetition (Face2rep, p = 0.25). Participants were exposed to these implicit contingencies in a behavioral session prior to scanning. In both the behavioral and the scanning sessions, they had to perform an orthogonal task on all stimuli. Individual left and right FFA and Occipital Face Area (OFA) were defined in an independent localizer task, and were used as regions of interest.

Critically, our design also allowed us to investigate brain responses to predicted face exemplars (Face2alt). This is important, because although Expectation Suppression has been widely documented in the early visual cortex^8, 26, 27^, evidence for Expectation Suppression for more complex visual stimuli such as individual objects or faces remains limited. Two functional Magnetic Resonance Imaging (fMRI) studies report category-specific Expectation Suppression in the Fusiform Face Area (FFA), which responded less strongly to faces when participants were expecting faces rather than houses^9, 11^. Yet, predicting the occurrence of a face in general (e.g. any face vs. any house) is a different process from predicting a particular face with specific features (e.g. John vs. Mary). Besides, it is to date unclear whether RS and its modulation by repetition probability reflect exemplar-specific predictions. The first face of a trial may induce the prediction of its own repetition in an exemplar-specific fashion, resulting in neural suppression when this prediction is fulfilled, but RS could also result from predictions about the event type (repetition or alternation). Recent studies indeed show that brain responses to the most likely event type are overall suppressed, orthogonally to repetition or alternation effects^24, 28^. Hence, studies investigating repetition probability effects on RS do not unambiguously show the existence of exemplar-specific predictions in the FFA, and whether this region codes for predictions about individual face exemplars is still an open empirical issue.

**Figure 1:**
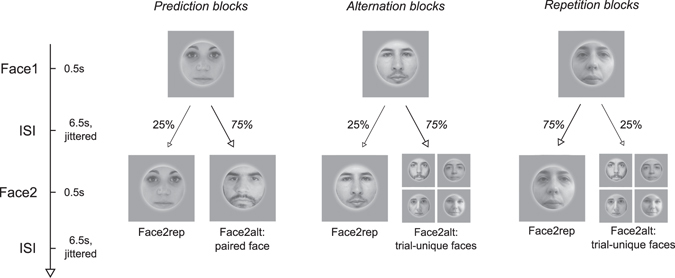
Overall experimental design. Each trial consisted of two faces, each presented for 0.5 s, and each followed by a long Inter-Stimulus Interval (ISI; 6.5 s, jittered). The first face (Face1) was either repeated (Face2rep, Repetition Trials) or followed by a different face (Face2alt, Alternation Trials) with variable contingencies depending on block type. Face2alt was specifically paired with Face1 in Prediction Blocks, while unpredictable and trial-unique in Repetition & Alternation Blocks. Participants were not informed of these contingencies. All faces were randomly tilted 1.5° clockwise or counterclockwise, which participants had to report by pressing a response button after the offset of each face.

In the present paradigm, alternations are predictable, and hence might lead to suppressed brain responses. Meanwhile, repetitions are less probable, and might under these circumstances lead to either enhanced or suppressed responses^25^. Importantly, to evaluate both effects, the FFA response to alternations and the FFA response to repetitions, we could not simply compare these two conditions, as was done in previous studies^14, 16–18^, which typically used short Inter-Stimulus Interval (ISI) between both images of a trial, and a single regressor for both images. Instead, we used long ISI between all faces, enabling us to assess separately brain responses to unpredicted faces (Face1), to surprising repetitions (Face2rep) and to expected alternations (Face2alt). This allowed us to use Face1 as a baseline, against which we could evaluate the direction (suppression vs. enhancement) of repetition and alternation effects independently. As a control, our design also included experimental blocks in which alternations involved unpredictable trial-unique faces, and in which repetitions were either likely (*P* = 0.75, ‘Repetition Blocks’) or unlikely (*P* = 0.25, ‘Alternation Blocks’) (Fig. 1, middle and right). This allowed us to control for the presence of RS between Face1 and Face2rep, and for its modulation by repetition probability, despite our non-classical setup with long ISI and without a clear pair structure.

## Methods

### Participants

Twenty-nine healthy right-handed adult volunteers (19 female, age 23.6 ± 3.0, mean ± SD) with normal or corrected-to-normal vision participated in this study. Data from one subject was excluded due to excessive head motion. All volunteers gave written informed consent to participate in this study.

### Ethics statement

All methods were carried out in accordance with relevant guidelines and regulations. The protocole was approved by the ethics committee “Comité de Protection des Personnes – Ile-de-France VI – Groupe Hospitalier Pitié-Salpêtrière” (ref: CPP/3-12 - ID RCB: 2011-A01618-33).

### Stimuli

Face stimuli were gray-scale, full-front digital photos of 16 recurrent faces, 124 trial-unique faces and 14 localizer faces (half female) used in a previous study^21^. House stimuli used in the functional localizer were gray-scale, full-front digital photos of 14 houses. All stimuli were fit behind a circular mask (radius = 4.2 deg), eliminating outer contours of the images. Stimuli were displayed centrally on a 50% grey background, using MATLAB (MathWorks, Natick, MA, USA) and the Psychophysics Toolbox^29^, on a Sun Microsystems L194RH screen (1024 × 786 resolution, 60 Hz refresh rate) in the behavioral session, and using a rear-projection screen using a luminance-calibrated EPSON Z8050 projector (1024 × 768 resolution, 60 Hz) in the fMRI session.

### Experimental task

Our experimental design is illustrated in Fig. 1. All faces were presented for presented for 0.5 s, and randomly tilted 1.5° clockwise or counterclockwise, which participants had to report by pressing a response button using their right hand (clockwise) or left hand (counterclockwise) after the offset of each face. Unbeknownst to participants, faces were arranged in trials, with the first face (Face1) either followed by a different face (Face2alt) or by a repetition of the same face (Face2rep). Each face was followed by a variable Inter-Stimulus Interval (ISI; 6.5 s, jittered, ±1 s for 15 participants and ±2 s for 14 participants), which allowed us to analyze brain responses to the Face1, Face2rep, and Face2alt separately. During this ISI, a fixation cross was presented, which alternated between darker and lighter after every trial. Participants completed 6 blocks of 48 trials (i.e. 96 faces) each, in separate 12-minute scanner runs.

Our design involved 3 types of blocks: “Prediction Blocks”, “Repetition Blocks” and “Alternation Blocks”. The order of these 3 types was defined for each participant (counterbalanced across participants), and applied to both blocks 1 to 3 and blocks 4 to 6. Participants were never explicitly instructed that different blocks used different probabilistic contingencies.

In “Prediction Blocks” (PB), there were 25% of repetitions and 75% of alternations, with predictable Face2alt. Specifically, PBs used 6 different faces (picked from the 16 recurrent faces) arranged in 3 pairs (1 male-female, 1 male-male and 1 female-female), and each face was 25% likely to repeat itself and 75% likely to be followed by its corresponding paired face.

In “Repetition Blocks” (RB), there were 75% of repetitions and 25% of alternations, and conversely for “Alternation Blocks” (AB), similarly to previous studies^14–18^. RB and AB respectively used 6 and 4 different faces (picked from the 16 recurrent faces) as Face1 and Face2rep, plus trial-unique faces as Face2alt, appearing only once during the experiment. This ensured that the 16 recurrent faces were presented roughly the same number of times across block types (PB: 6 faces presented 32 times each, RB: 6 faces presented 28 times each, AB: 4 faces presented 30 times each).

The fMRI session was preceded by a behavioral training session on the day before, so that participants would implicitly learn the pairs of the Prediction Blocks. The design was identical to that used in the scanner, except for two differences. First, ISIs were shorter (2 s instead of 6.5 s in the scanner). Second, in order to emphasize the pair structure during learning, stimuli were presented along with a square frame that was darker/lighter during even/odd pairs of faces, in accordance with the color of the fixation cross.

Eye position was monitored using a camera-based infrared eyetracker (Sensomotoric Instruments, Berlin, Germany). Although the quality of this recording was not sufficient to allow for a formal analysis of these signals, the eye signals were monitored by the experimenter during task execution to ensure adequate fixation.

### Localizer task

Regions of interests (ROI) were defined from a separate scanning run of 500 s, divided into 20 blocks which involved either 14 faces or 14 houses (stimulus duration: 750 ms, ISI: 250 ms), and a 11 s rest period. Participants had to detect immediate stimulus repetitions, which they reported by pressing any of the response buttons. The localizer run was acquired at the end of the experiment, after the task runs.

### fMRI data acquisition and preprocessing

Functional images were acquired using a 3 T Verio MRI system (Siemens, Erlangen, Germany) with a T2*-weighted multi-band accelerated EPI sequence (TR/TE = 1200/29 ms, 42 transversal slices, voxel size 2 × 2 × 2 mm, 69° flip angle, multi-band acceleration factor: 3). Anatomical images were acquired with a T1-weighted MP-RAGE sequence (TR/TE = 2300/4.18 ms, voxel size 1 × 1 × 1 mm, 9° flip angle). The first six volumes of each block were discarded to allow T1 equilibration. We used SPM8 (Wellcome Trust Centre for Neuroimaging, London, UK) for realignment of functional images to the mean image, slice timing, coregistration with the participant’s anatomical image, normalization to the MNI-152 space, and spatial smoothing (Gaussian kernel: 8 mm FWHM).

### Statistical analysis

We used a generalized linear model (GLM) to account for the fMRI data in each participant. Our model included stick-function regressors coding for onsets and durations of each face separately.

On most trials, Face1 was unrelated to the Face2 presented during the preceding trial. Nonetheless, as our trial sequence was fully randomized, on certain trials, Face1 could be a repetition of the Face2 from the previous trial. Our analysis modeled these images using a separate regressor, ‘Face1rep’, in all blocks. Besides, in PB, Face1 could also be the face associated to the Face2 of the previous trial. Our analysis modeled these images using a separate regressor, ‘Face1pred’, in PB only. The regressor named ‘Face1’ corresponded to the Face1 images that were neither Face1rep nor Face1pred.

Our model included 5 stick-function regressors coding for onsets and durations of Face1, Face1rep, Face1pred, Face2rep and Face2alt images, and an additional regressor coding for the onset and amplitude of response times. These regressors were convolved with the canonical hemodynamic response function (HRF). We modeled experimental blocks using separate regressors and a constant term for each block. Motion parameters were included as nuisance variables.

After estimation of the model, the effect of face repetition was computed as the difference between Face2rep and Face1, and the effect of face alternation as the difference between Face2alt and Face1. Negative (resp. positive) effects here indicate suppressed (resp. enhanced) responses to Face2 relative to Face1. Because the faces used as Face2alt in RB and AB are trial-unique and novel, the corresponding regressor confounds novelty and alternation effects. The comparison between Face2alt and Face1 responses is however of particular interest in our Prediction Blocks, where it quantifies expectation suppression. Simple ANOVAs and t-tests across subjects were then used at the second level. Results were analyzed using MATLAB and R statistical software. Statistical tests are two-tailed.

### ROI Definition

We used our localizer task to define two face-sensitive bilateral regions of interest (ROI): the Fusiform Face Area (FFA_L_, FFA_R_) and the Occipital Face Area (OFA_L_, OFA_R_) (L and R subscripts indicate the left and right hemispheres). From a GLM using regressors coding for face and house blocks, we computed a [Face - House] contrast for each participant, and defined our ROIs as 4-mm–radius spheres centered on local maxima of this t-statistic map around the anatomically defined fusiform gyrus. The mean (±s.e.m.) MNI coordinates were [−42 ± 1, −51 ± 1, −20 ± 1] (FFA_L_, n = 27), [43 ± 1, −48 ± 2, −20 ± 1] (FFA_R_, n = 28), [−42 ± 1, −76 ± 2, −11 ± 1] (OFA_L_, n = 25), and [45 ± 1, −73 ± 2, −10 ± 1] (OFA_R_, n = 26). Finally, for completeness, we defined an “early visual” ROI at the group-level, using an anatomical mask around the calcarine sulcus, which we refer to as ‘V1’. Because many outlying values were found in the right V1 data, we only analysed responses from left V1.

## Results and Discussion

We report and discuss 3 analyses. First, we focused on Repetition Blocks and Alternation Blocks to evaluate whether repetition suppression and repetition probability effects could be replicated with the conditions of our paradigm. Second, focusing on the Prediction Blocks, we evaluated exemplar-specific expectation suppression for faces that were predicted by a distinct but associated face. Third, we tested the hypothesis that repetition suppression can be turned into repetition enhancement in our Prediction Blocks. These analyses were conducted on individually defined FFAs and OFAs, and on a group-defined V1 region of interest.

### Repetition Suppression and repetition probability effects in face-sensitive regions

**Figure 2:**
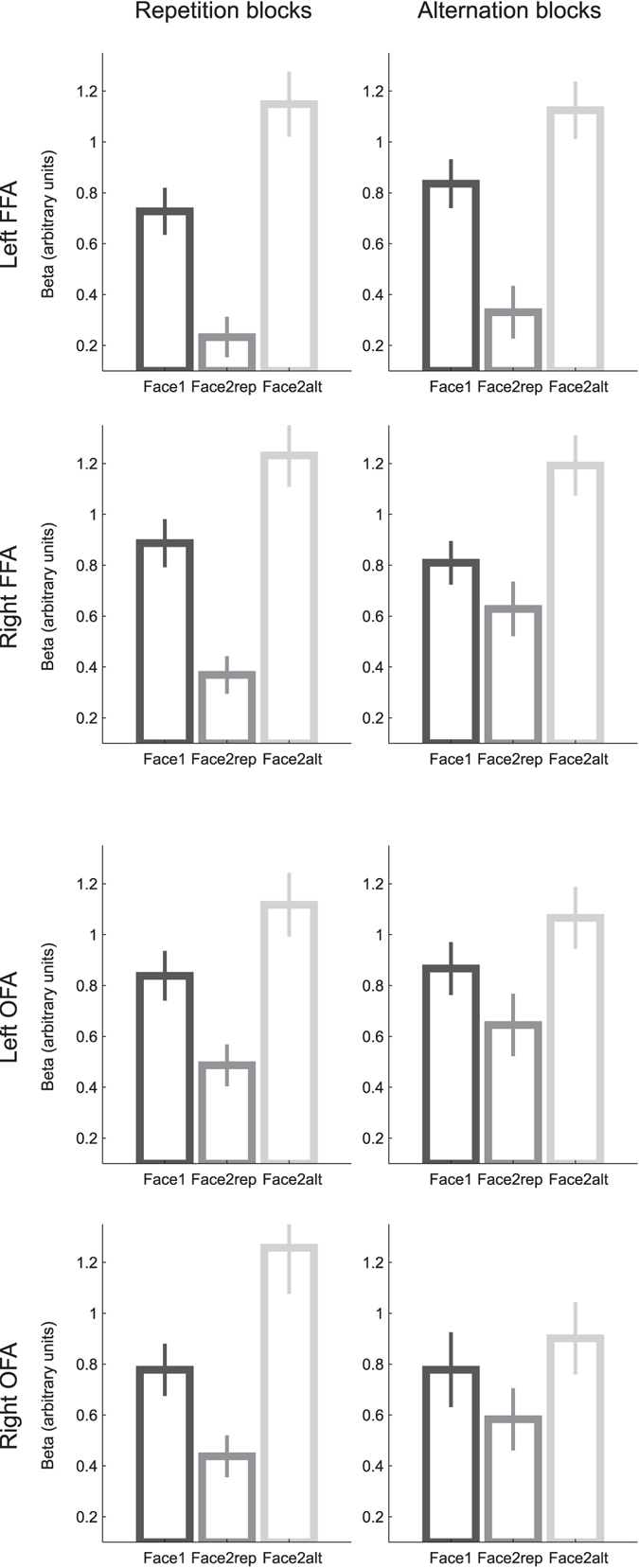
Parameter estimates (mean ± s.e.m. across 28 participants) for Face1, Face2alt and Face2rep, in the left FFA, right FFA, left OFA, and right OFA, during Repetition Blocks (left) and Alternation Blocks (right).

First, to ensure that our design yielded RS despite our long ISI (6.5 s, jittered) and the repeated use of a small set of face stimuli, we analyzed FFA and OFA responses in Repetition Blocks, during which RS was expected to be the strongest. We ran separate 2-way ANOVAs for each ROI, with hemisphere and repetition (Face1 vs. Face2rep) as within-participant factors. We found significant repetition effects in the FFA (F_(1,26)_ = 107.9, p = 1e-10; Fig. 2), in the OFA (F_(1,25)_ = 30.2, p = 1e-5), and in V1 (F_(1,27)_ = 5.69, p = 0.025) with reduced responses for Face2rep compared to Face1 images. In our design, thus, fMRI responses to the repeated presentation of a face were reduced in comparison with the first presentation of this same face.

Previous studies have reported a modulation of the amplitude of RS by repetition probability, using short ISI between both faces of a trial, and hence, a single regressor for each trial^14, 16–18^. We assessed whether this modulation was present in our design by considering the 3-way ANOVA with repetition probability (Repetition Block vs. Alternation Block), repetition effect (Face1 vs. Face2rep) and hemisphere as within-participant factors. In the FFA (Fig. 2, top 4 graphs), the repetition effect significantly interacted with repetition probability and hemisphere (F_(1,26)_ = 15.0, p = 6e-4). Analyzing both hemispheres separately, we found that the critical Repetition × Repetition Probability interaction was significant in the right FFA (F_(1,27)_ = 5.8, p = 0.024), but not in the left FFA (F_(1,26)_ = 8e-2, p = 0.93). The modulation of RS by repetition probability was also absent in the OFA (Fig. 2, bottom 4 graphs, Repetition × Repetition Probability interaction: F_(1,25)_ = 1.2, p = 0.29) and in V1 (F_(1,27)_ = 0.14, p = 0.71).

Please note that in the present design, RS is assessed by comparing Face1 and Face2rep, and not by comparing repetition and alternation trials as in traditional designs with short ISI. Indeed, in both Repetition Blocks and Alternation Blocks, Face2alt were trial-unique (i.e. novel) faces, while Face1 and Face2rep were familiar faces that were presented recurrently during the experiment. Hence, enhanced neural responses to these trial-unique faces are probably a novelty effect, so these activations cannot be compared to the others, and they are only displayed in Fig. 2 for completeness.

In short, we replicated previously observed RS (in the FFA, OFA, and V1) and its modulation by repetition probability (in the right FFA only), despite a stimulus presentation rate much slower than in previous studies documenting this effect^14, 16–18^, which extends the scope of past results.

### Expectation Suppression for predictable alternations

**Figure 3:**
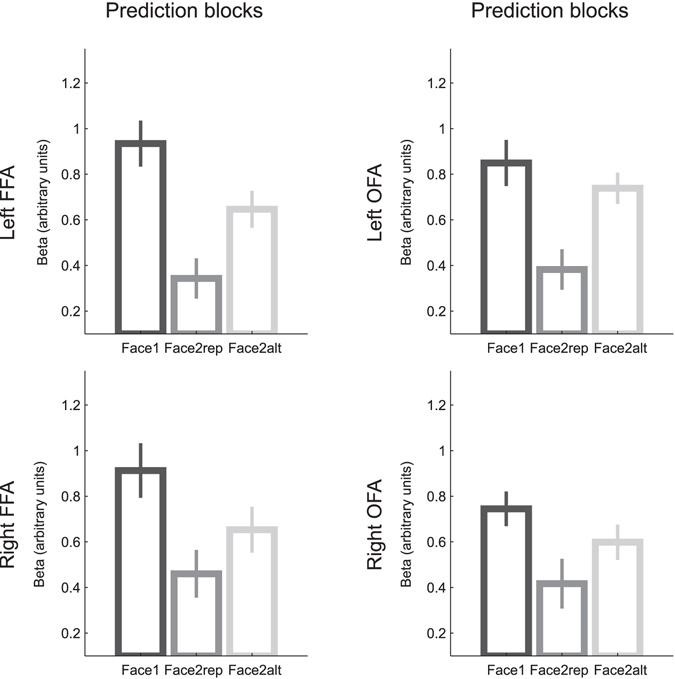
Parameter estimates (mean ± s.e.m. across 28 participants) for Face1, Face2alt and Face2rep, in the left and right FFA and OFA, during Prediction Blocks.

We then investigated Expectation Suppression in our design, with the following reasoning: if the FFA encodes exemplar-specific predictions, it should show reduced responses to predictable Face2alt images compared with unpredicted Face1 images, i.e. Expectation Suppression. This is indeed what we found: a 2-way ANOVA with hemisphere and predictability (Face1 vs. Face2alt) as factors revealed significant suppression of FFA responses to predictable Face2alt images (F_(1,26)_ = 11.6, p = 0.002; no interaction with hemisphere: F_(1,26)_ = 0.1, p = 0.79) (Fig. 3). This effect was absent in the OFA (F_(1,25)_ = 2.4, p = 0.13), with a marginally significant interaction (F_(1,24)_ = 4.2, p = 0.052) between region (FFA vs. OFA) and Expectation Suppression (Face1 vs. Face2alt). Expectation Suppression was also marginal in our V1 region of interest (F_(1,27)_ = 3.4, p = 0.07). The reduced response to predictable Face2alt relative to unpredictable Face1 images observed in the FFA demonstrates the existence of prediction signals for individual face exemplars. Expectation Suppression can be interpreted in terms of predictive coding, as a reduction of prediction errors for correctly predicted inputs.

Can we rule out alternative accounts of our results? First, familiarity could not be a confounding factor, since Face1 and Face2alt images had been presented the same number of times to each participant. Second, it is very unlikely that participants attended more strongly to Face1 than to Face2alt. Indeed, the pair structure was not explicit to participants, who performed a tilt discrimination task on each image, with similar ISI between both images of each trial and between trials, and no cue signaling the beginning of a trial. Furthermore, Face1 and Face2alt images differed neither on performance (87.1 ± 1.5% vs. 86.6 ± 1.6% of correct responses; W = 404, p = 0.85, Wilcoxon rank sum test) nor on response times (437 ± 47 ms vs. 442 ± 45 ms; t_(27)_ = 0.43, p = 0.67, paired T-test) (see also Supplementary Fig. 1). Besides, we found that Expectation Suppression also occurred for Face1 images, when considering the trials where (by chance) Face1 was predictable from the second face on the previous trial (main effect of Face1 vs. Face1pred: F_(1,26)_ = 6.5, p = 0.02, 2-way hemisphere x regressor ANOVA; Face1pred corresponds to 5% of the trials). In other terms, Expectation Suppression was present independently from the position of the predicted face in the sequence, thus ruling out an account of our results in terms of stronger attention on Face1. Third, we can also discard an alternative interpretation based on cross-adaptation, that is, the decreased response to the second stimulus of a pair, when both stimuli are different but activate the same neuron^30, 31^. Indeed, in our design cross-adaptation may reduce responses to Face2alt images, but if so it would similarly reduce responses to Face1 images, which are preceded by another face with the same delays. Thus, cross-adaptation could actually dampen our effect, but it could not explain it. Finally, we ruled out a possible effect of gender congruency between both faces of a pair, by computing Expectation Suppression separately for the pairs that involved two faces with the same gender (female-female or male-male) and different genders (female-male). We found significant Expectation Suppression in both the first case (F_(1,26)_ = 6.6, p = 0.016) and the second case (F_(1,26)_ = 9.9, p = 0.004), which demonstrates that the presence of Expectation Suppression in our data cannot be attributed to the same-gender pairs.

The reduction in neural response to expected inputs, hypothesized by predictive coding^6, 7^ and known as expectation suppression, has been highlighted in the early visual cortex by several studies using cues to guide participants’ expectations^8, 26, 27, 32, 33^. Evidence for Expectation Suppression with more complex stimuli is sparser. Two studies in humans demonstrate expectation suppression in the FFA when participants expected faces rather than houses^9, 11^, thereby showing that the human FFA codes for predictions at the level of the stimulus category. A similar phenomenon was reported in macaque monkeys: after extensive exposure to transitions between objects (13 sessions & 42 sessions for the two monkeys in this study), individual neurons in the inferotemporal cortex exhibited reduced responses to stimuli following (vs. violating) the “learned” transitions^10^. However, since the stimuli used in this study belonged to different categories, the observed expectation suppression could reflect category-level expectations, rather than stimulus-specific expectations. We acknowledge that a similar paradigm, simpler than ours, with learnt transitions and violations without repetitions, would also be appropriate to provide evidence for exemplar-specific expectation suppression in the human temporal cortex (although it would leave open the question of surprising repetitions addressed below). Still, to the best of our knowledge, the present study is the first to demonstrate expectation suppression for specific face exemplars in the FFA, and more generally for specific elements within a relatively homogeneous category of stimuli. Predicting a face in general, as opposed with another object category, is a different process from predicting a specific face with its particular features as opposed with another specific face. Thus, category-specific and exemplar-specific expectations in the FFA may rest on different neuronal populations. Further research is needed to clarify this issue.

### Neural responses to surprising repetitions

Our third analysis addressed the recent hypothesis that unlikely repetitions presented along with predictable alternations might become so surprising that they elicit enhanced brain responses (Repetition Enhancement)^25^, rather than suppressed responses as classically observed for repeated inputs (RS)^3, 4^. Our data disconfirms this proposition: in Prediction Blocks, we found reduced responses for Face2rep compared to Face1 in both the FFA and the OFA (F_(1,26)_ = 22.3, p = 7e-5; and F_(1,25)_ = 14.3, p = 9e-4 respectively, see Fig. 3), and no difference in V1 (F_(1,27)_ = 0.87, p = 0.36). In other words, although Expectation Suppression shows that the brain could learn the contingencies and predict the forthcoming face exemplar, this manipulation could not turn RS into repetition enhancement.

We note that previous studies^14–19^ did not find repetition enhancement for unlikely repetitions. However, the use of trial-unique faces during alternation trials might have created a situation in which repetitions are still easier to predict than alternation, even when less likely, because the identity of the second face of alternation trials is impossible to predict. In our ‘Prediction Blocks’, participants implicitly developed face-specific predictions about an alternative stimulus (as evidenced by expectation suppression), which was three times more likely to occur than a repetition. Critically however, even in these conditions we still found suppressed FFA responses for unlikely repetitions. In fact, a post-hoc comparison indicated that FFA responses were more suppressed for these surprising repetitions than for the predictable alternations (F_(1,26)_ = 5.838, p = 0.023).

This ‘unsuppressible’ suppression is in line with the view that the modulation of RS by repetition probability results from independent effects of repetition and expectation^15, 24, 28, 34^. Accordingly, recent views^24, 35^ propose that RS rests on low-level expectations based on local transition probabilities^36, 37^ (which could be equivalent to a predictive account of neuronal adaptation), while its modulations by repetition probability would rely on hierarchically higher expectations. Just like neuronal representations are adapted to the statistics of our visual environment^38^, a ‘default’ repetition prior at the local level, possibly hard-wired in the neuronal architecture, would be adapted to the relative stability of our perceptual environment across short timescales^39–41^. Such a prior could be embodied through synaptic depression (fatigue model), synaptic potentiation (facilitation model), lateral inhibition (sharpening model), predictive coding mechanisms (Bayesian ‘explaining away’ model)^5, 12^ or a combination of these. Whether this putative low-level ‘default’ repetition prior could be reversed by more extensive training than in the present study, like the light-from-above prior^42^, up to the point where RS turns to enhancement as recently hypothesized by Segaert and colleagues^25^, remains to be investigated.

### Conclusion

Using a probabilistic context in which certain faces specifically predicted the occurrence of others, we demonstrated that the FFA exhibits exemplar-specific Expectation Suppression. We also found that in this context, where repetitions are surprising, Repetition Suppression is still maintained in the FFA, which supports the view that repetition effects cannot be fully modulated by expectations, and hence that repetition and expectation effects rest on independent mechanisms. Altogether, this provides new empirical evidence about the nature of predictive signals in the human extrastriate cortex.

## Electronic supplementary material

Supplementary Material
