# Supplementary material for: Unsuppressible Repetition Suppression and exemplar-specific Expectation Suppression in the Fusiform Face Area
Source: Sci Rep. 2017 Mar 13;7:160. doi: 10.1038/s41598-017-00243-3 (PMC5428004; doi:10.1038/s41598-017-00243-3)
Supplement: Supplementary file 1 — Supplementary Material [file 41598_2017_243_MOESM1_ESM.pdf]

Unsuppressible Repetition Suppression and exemplar-specific Expectation Suppression in the Fusiform Face Area

Auréliane Pajani[1], Sid Kouider[1], Paul Roux[2,3,4], Vincent de Gardelle[5,6]

[1] Brain and Consciousness group (ENS, EHESS, CNRS), Département d'Études Cognitives, École Normale Supérieure - PSL Research University, 75005 Paris, France

[2] Service Universitaire de Psychiatrie d'adultes, Centre Hospitalier de Versailles, 78157 Le Chesnay, France

[3] Laboratoire HandiRESP, EA4047, Université Versailles Saint Quentin en Yvelines, 78000 Versailles, France

[4] Fondation Fondamental, Créteil, France

[5] Centre d'Economie de la Sorbonne, CNRS & Université Paris 1, 75013 Paris, France,

[6] Paris School of Economics, 75014 Paris, France

Correspondence: Vincent de Gardelle

Maison des Sciences Economiques, 106-112 Boulevard de l'Hôpital, 75647 Paris Cedex 13

vincent.gardelle@gmail.com

Supplementary Figure 1

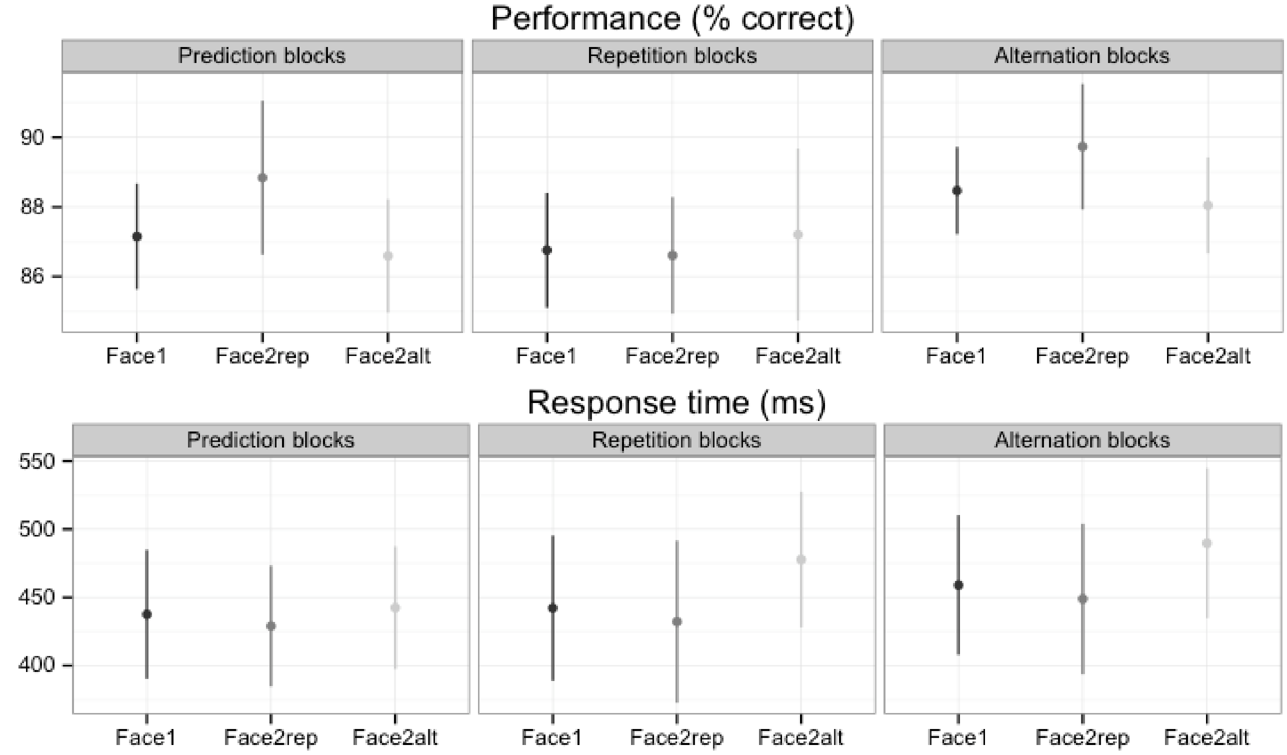

Average performance (% of correct responses) and response times (ms) across participants (mean ±s.e.m. across 28 participants), for the different image types (Face1, Face2rep and Face2alt), and block types (Prediction Blocks, Repetition Blocks and Alternation Blocks).

Separate Kruskal-Wallis rank sum tests indicated no effect of block type ($H_{(2)}$=0.47, p=0.79), or image type ($H_{(2)}$=0.37, p=0.83) on the proportion of correct responses. Regarding response times (RTs), a 2-way ANOVA with block type and image type as within-participant factors showed no main effect of block type ($F_{(2,54)}$=1.7, p=0.19), but a main effect of image type ($F_{(2,54)}$=5.2, p=0.008), with no interaction ($F_{(4,108)}$=0.8, p=0.90). These results suggest that attentional levels were similar across blocks. Post-hoc analyses also suggest that the main effect of image type may correspond to a novelty/familiarity effect. Indeed, slower responses to Face2alt images were found in Repetition Blocks ($F_{(2,54)}$=4.4, p=0.017) and Alternation Blocks ($F_{(2,54)}$=3.4, p=0.042), where these images are trial-unique (i.e. presented only once during the experiment). In Prediction Blocks, in which all image types involved stimuli with equal novelty/familiarity, RTs did not seem to be affected by image type ($F_{(2,54)}$=0.4, p=0.70).
